# Facile modulation the sensitivity of Eu^2+^/Eu^3+^-coactivated Li_2_CaSiO_4_ phosphors through adjusting spatial mode and doping concentration

**DOI:** 10.1038/s41598-020-77185-w

**Published:** 2020-11-19

**Authors:** Luhui Zhou, Peng Du, Li Li

**Affiliations:** 1 Department of Microelectronic Science and Engineering, School of Physical Science and Technology, Ningbo University, Ningbo, 315211 Zhejiang China; 2 Schol of Science, Chongqing University of Posts and Telecommunications, Chongqing, China

**Keywords:** Materials science, Optics and photonics

## Abstract

Series of Eu^2+^/Eu^3+^-coactivated Li_2_CaSiO_4_ phosphors were prepared by solid-state reaction technique. All the samples emitted the unique emissions of Eu^2+^ and Eu^3+^ ions when excited by 395 nm, while the strongest emission intensity was received when *x* = 0.03. On the basis of theoretical discussion, it is evident that crossover relaxation should be responsible for the thermal quenching mechanism which was further proved by the unchanged lifetime at elevated temperature. Besides, through analyzing the inconsistent responses of the emission intensities of the Eu^2+^ and Eu^3+^ ions to the temperature, the optical thermometric properties of the designed phosphors were studied. By selecting different emissions of Eu^3+^ ions and combining with that of the Eu^2+^ ions, adjustable sensitivities were realized in the resultant phosphors. Furthermore, the sensitivities of the studied compound were also found to be greatly affected by the doping concentration. The maximum absolute and relative sensitivities of the synthesized compounds were 0.0025 K^−1^ and 0.289% K^−1^, respectively. These achieved results implied that the Eu^2+^/Eu^3+^-coactivated Li_2_CaSiO_4_ phosphors were promising candidates for optical thermometry. Additionally, this work also provided promising methods to modulate the sensitivities of the luminescent compounds by adjusting spatial mode and doping concentration.

## Introduction

Temperature, acts as a fundamental thermodynamic constant, has drawn considerable interest since it plays a significant role in our daily life, industrial manufacture, medical treatment and scientific research. Thus, its accurate measurement with high resolution is required. Unfortunately, the widely used traditional thermometers (e.g., liquid-in-glass and thermocouple thermometers) suffer from shortages of low accurate, unsatisfied resolution and contact characteristics. To fulfill these drawbacks, contactless optical thermometer, which shows the advantages of high spatial resolution, high accurate, fast response, remote monitoring, etc., had been developed and attracted intensive attention^1–3^. Generally, the contactless optical thermometers are realized by employing the fluorescence intensity rate (FIR) technique to study the diverse responses of the emission intensities of the thermally coupled levels (TCLs) to the temperature^4,5^. In order to achieve an ideal optical temperature sensing materials by using the FIR technique, the luminescent materials should exhibit two distinct emission peaks, which must have different changing tendency to the temperature, as the monitoring signals. Currently, the thermometric behaviors of the rare-earth ions, such as Er^3+^, Tm^3+^, Nd^3+^, etc., have been widely studied since they have pairs of TCLs^6–9^. Note that, the energy separation of the TCLs is narrow (200–2000 cm^−1^) which hinders its maximum relative sensitivity (*S*_*r*_) value as well as induces the deviation of the experimental FIR value from the real value, resulting in large error. To overcome these kinds of intrinsic imperfections, novel optical thermometry based on dual-emitting centers was proposed. Up to date, some results have been reported in the dual-emitting centers based optical thermometers, such as Eu^3+^/Tb^3+^, Eu^3+^/Mn^2+^, Ce^3+^/Tb^3+^, Eu^2+^/Eu^3+^ and Bi^3+^/Eu^3+^ coactivated luminescent materials^10–14^. Notably, for practical applications, the developed optical thermometers should exhibit large sensitivities, whereas the currently reported sensitivities of the dual-emitting centers based optical thermometers were still not high enough. Thus, some available routes should be carried out to further improve the thermometric performance of the dual-emitting centers based optical thermometers.

As a part of rare-earth ions, trivalent Eu^3+^ ions have been intensively researched as red-emitting activators because of its intense sharp emissions originating from the ^5^D_0_ → ^7^F_J_ (J = 1, 2, 3 ,4)^15,16^. Moreover, the bivalent Eu^2+^ ions are also regarded as vital luminescent activators since it can emit abundant emissions (i.e., from ultraviolet to red) from the 4f ground state to the 5d excited level^17,18^. Note that, the probabilities of the intra-4f transitions of Eu^3+^ ions and the 4f–5d transitions of Eu^2+^ ions are all significantly impacted by the crystal field of the host. Therefore, choosing proper luminescent host is a facile pathway to improve the optical properties of Eu^2+^ and Eu^3+^ ions. On the other hand, it was also demonstrated that the emission intensities of the Eu^2+^ and Eu^3+^ ions exhibited diverse thermal-dependent emission intensity behaviors^19,20^. As a consequence, the Eu^2+^/Eu^3+^-coactivated luminescent materials were able to exhibit the thermometric properties and showed promising applications in contactless optical thermometers. However, in previous reports, only the photoluminescent and thermometric properties of the Eu^2+^/Eu^3+^-coactivated compounds were studied, whereas the research on how to improve the thermometric behaviors of the Eu^2+^/Eu^3+^-coactivated compounds is still not enough. Therefore, it would be very interesting to search for some effective methods to improve the thermometric properties of the rare-earth ions activated optical materials.

In this work, we selected the Li_2_CaSiO_4_ as the luminescent host owing to its high thermal stability. Besides, it was also proved that the Eu^2+^-activated Li_2_CaSiO_4_ phosphors can emit broad blue emission upon near-ultraviolet light excitation^21^. Evidently, the blue emission of the Eu^2+^ ions was totally separated from featured red emissions of Eu^3+^ ions. As a result, optical thermometry is expected to be realized in the Eu^2+^/Eu^3+^-coactivated Li_2_CaSiO_4_ phosphors by using the FIR technique. Herein, series of the Eu^2+^/Eu^3+^-coactivated Li_2_CaSiO_4_ phosphors were synthesized by the simple solid-state reaction technique. The phase compositions, morphology, decay time and photoluminescent properties of the prepared samples were investigated. Furthermore, on the basis of the temperature-dependent lifetime along with the energy level diagram, the thermal quenching mechanism was studied. Additionally, the effects of the different emission combinations of Eu^2+^/Eu^3+^ ions and doping concentration on the sensitivities of the resultant phosphors were studied by means of the FIR technology.

## Results and discussion

The X-ray diffraction (XRD) profiles of the Li_2_CaSiO_4_:*x*Eu^2+^/Eu^3+^ phosphors were monitored so as to identify their phase components. As demonstrated in Fig. S1, when the dopant content was less than 2 mol%, the recorded diffraction peaks of the designed phosphors were the same as those of the standard tetragonal Li_2_CaSiO_4_ (JCPDS#27-0290), suggesting that the prepared phosphors had tetragonal phase and the Ca^2+^ ions can be replaced by the dopants. Nevertheless, several tinny impurity peaks originating from Eu_3_SiO_7_ (JCPDS#20-0404) occurred when the doping concentration was further increased (see Fig. S1). These results suggested that the dopants (i.e., Eu^2+^ and Eu^3+^) had a limited solid solubility in the Li_2_CaSiO_4_ host lattices.

**Figure 1:**
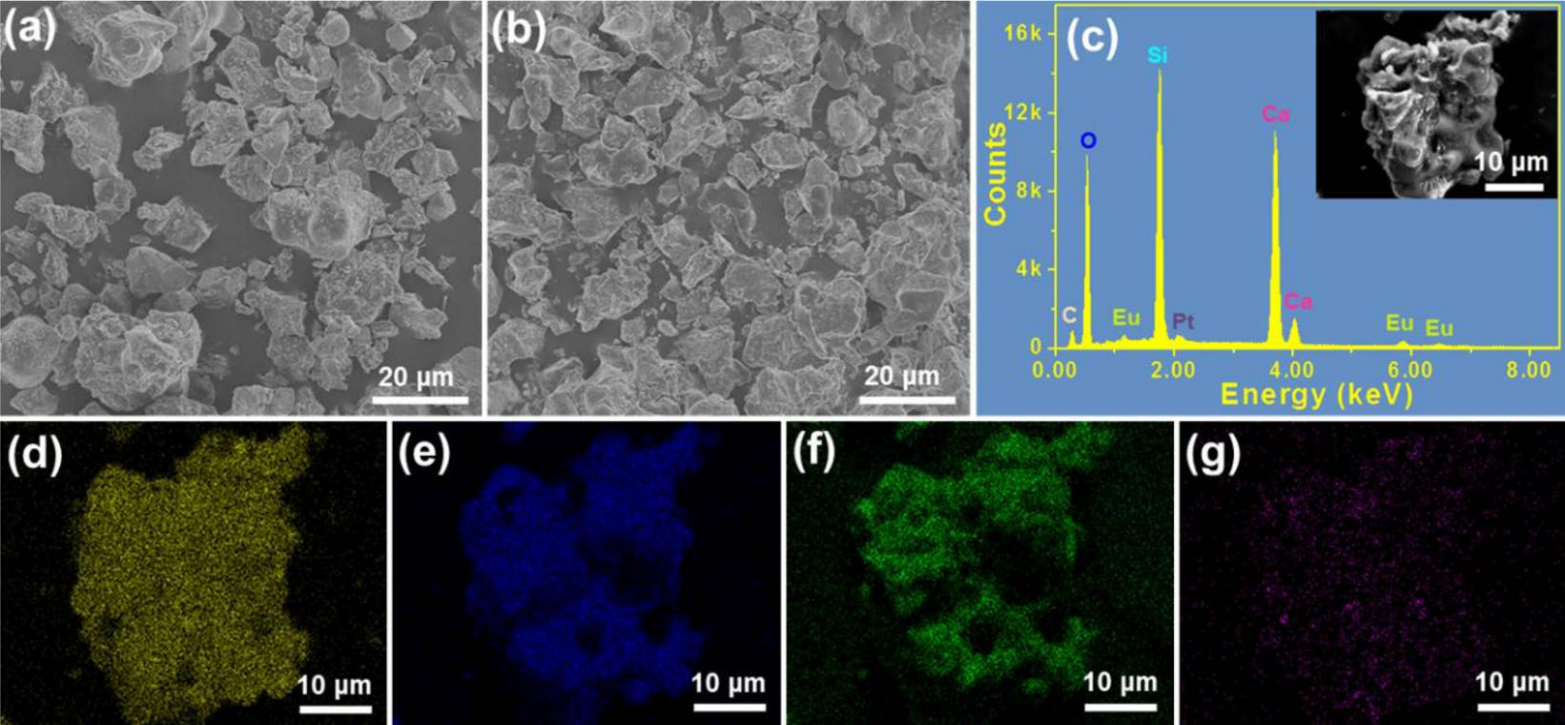
FE-SEM images of (**a**) Li_2_CaSiO_4_:0.005Eu^2+^/Eu^3+^ and (**b**) Li_2_CaSiO_4_:0.03Eu^2+^/Eu^3+^ phosphors, (**c**) EDX spectrum and (**d**–**g**) Elemental mapping of Li_2_CaSiO_4_:0.03Eu^2+^/Eu^3+^ phosphors.

For the sake of disclosing the morphological information of synthesized samples, the FE-SEM images of the representative Li_2_CaSiO_4_:0.005Eu^2+^/Eu^3+^ and Li_2_CaSiO_4_:0.03Eu^2+^/Eu^3+^ phosphors were detected, as shown in Fig. 1a,b, respectively. It can be seen that the studied compounds were made up of anomalous particles and their sizes were in micron level. Notably, although the doping content was changed, the morphology (i.e., size and shape) of the resultant products changed little, revealing that the introduction of the Eu^2+^/Eu^3+^ ions exhibited scarcely impact on the microstructure of the studied samples. Furthermore, according to the EDX spectrum (see Fig. 1c), one knows that that the synthesized samples contained the elements of Ca, Si, O and Eu. With aid of the EDX technique, we can not detect Li since it belongs to the light element with small K*α* energy. Additionally, the observation of the C peak in the EDX spectrum was assigned to the conductive tape, while the detection of the Pt peak in the EDX spectrum was attributed to the platinum electrode that was used for FE-SEM operation. Ultimately, it was also found that these detected elements (i.e., Ca, Si, O, Eu) were equally distributed throughout the particles, as presented in Fig. 1d–g.

As discussed above, the components of the studied samples can not be successfully confirmed by utilizing the EDX technique due to its limitation. For the purpose of getting deeper insight into the compositions and elemental states of the studied samples, the X-ray photoelectron spectroscopy (XPS) measurement was performed and the typical results of the Li_2_CaSiO_4_:0.03Eu^2+^/Eu^3+^ phosphors are depicted in Fig. S2. As described in Fig. S2a, only one peak centered at 57.8 eV, which was attributed to the Li^+^ 1 s, was observed in the high-resolution XPS spectrum^22^. The XPS spectrum presented in Fig. S2b was dominated by an intense peak with the binding energy of 348.7 eV which was ascribed to the Ca^2+^ 2p_3/2_^22^. The existence of the Si^4+^ 2p_3/2_ in the resultant compounds was confirmed by the binding energy at approximately 104.8 eV, as displayed in Fig. S2c^23^. Furthermore, the peak with the binding energy of around 532.4 eV was assigned to the O^2−^ 1 s (see Fig. S2d)^5^. In addition, the XPS spectrum consisted of two bands with the binding energies of 1134.5 and 1164.7 eV which were associated with the Eu^3+^ 3d_5/2_ and Eu^2+^ 3d_5/2_, respectively, as shown in Fig. S2e^24^. The XPS results did not only prove that the synthesized samples were composed of Li, Ca, Si, O and Eu elements, but also revealed that the Eu^3+^ ions were partly transferred into Eu^2+^ ions.

The diffuse reflectance spectrum of the Li_2_CaSiO_4_:0.03Eu^2+^/Eu^3+^ phosphors was measured to examine the absorption ability of the studied samples. As demonstrated in Fig. S3a, both the broad absorption band originating from the Eu^2+^ ions and a sharp peaks centered at 395 nm (^7^F_0_ → ^5^L_6_) arising from the Eu^3+^ ions were observed in the diffuse reflectance spectrum^21,25^, which further verified the coexistence of Eu^3+^ and Eu^2+^ ions in the resultant products. Furthermore, it has been confirmed that the relation between the absorption constant (i.e., *α*) and energy band gap (i.e., *E*_*g*_) keeps to the following function^26,27^:

1 $$\alpha hv=A{\left(hv-{E}_{g}\right)}^{n}$$

where *hv* refers to the energy, *A* is related to the coefficient, while the value of *n* can be 1/2, 2, 3/2 and 3 which corresponds to direct, indirect, forbidden direct, and forbidden indirect electronic transitions, respectively. Furthermore, the absorption spectrum (i.e., *F*(*R*)) is able to be achieved from the diffuse reflectance spectrum with the aid of the Kubelka–Munk expression, as defined below^28^:

2 $$F\left( R \right) = \left( {1 - R} \right)^{2} /2R,$$

here *R* is assigned to the reflectivity of the compounds. Through combining Eqs. (1) and (2), the following function is obtained:

3 $${\left[hvF\left(R\right)\right]}^{1/n}=A\left(hv-{E}_{g}\right),$$

For Li_2_CaSiO_4_, the *n* value is 1/2^29^. Thus, the *E*_*g*_ value of the Li_2_CaSiO_4_:0.03Eu^2+^/Eu^3+^ phosphors was demonstrated to be 4.54 eV through extrapolating the linear fitted region to [*hvF*(*R*)]^2^ = 0, as displayed in Fig. S3b.

**Figure 2:**
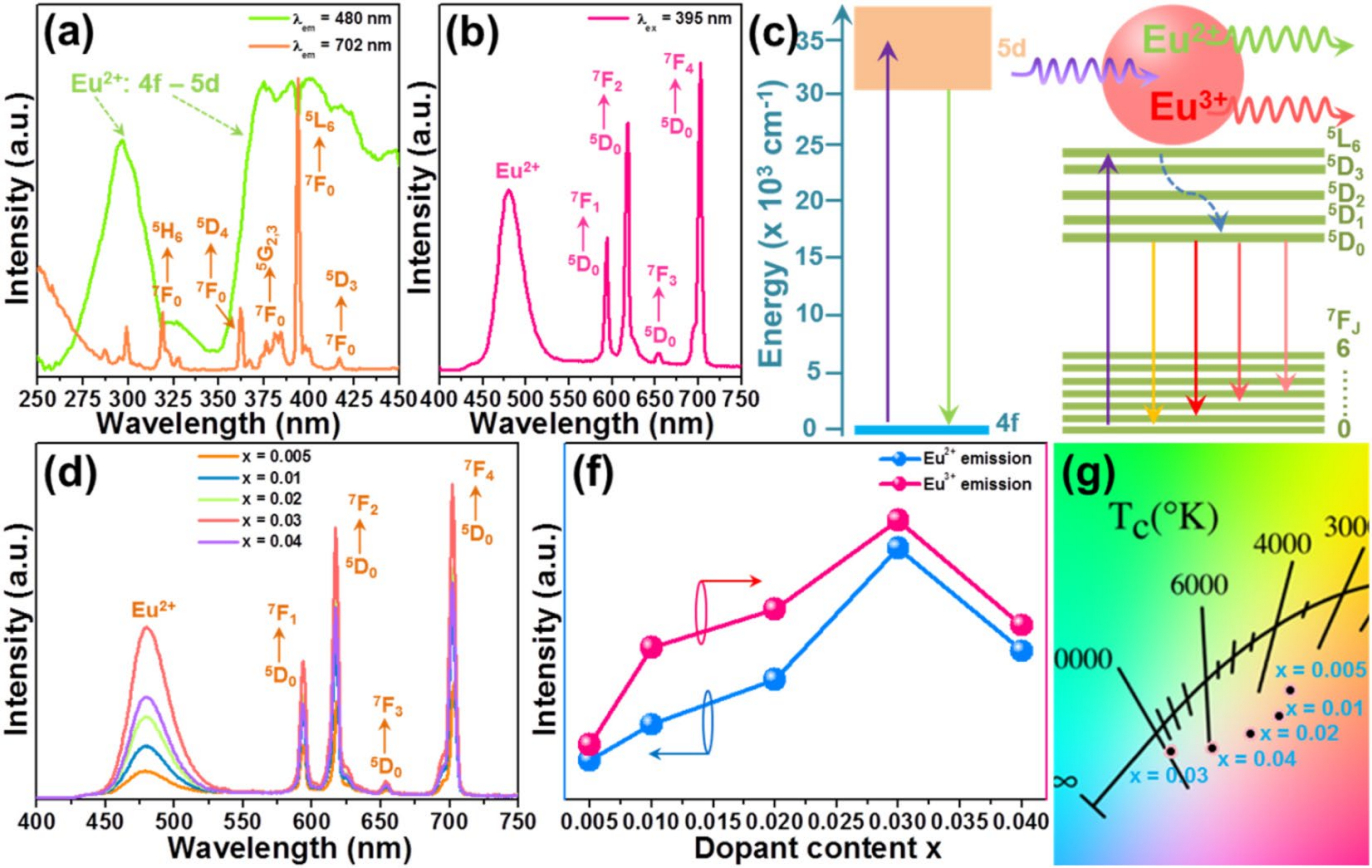
(**a**) Excitation and (**b**) Emission spectra of the Li_2_CaSiO_4_:0.03Eu^2+^/Eu^3+^ phosphors. (**c**) Energy level diagram of Eu^3+^ and Eu^2+^ ions. (**d**) Emission spectra of the Li_2_CaSiO_4_:*x*Eu^2+^/Eu^3+^ phosphors excited at 395 nm. (**f**) Emission intensity of the Li_2_CaSiO_4_:*x*Eu^2+^/Eu^3+^ phosphors at different doping contents. (**g**) CIE coordinate diagram of the Li_2_CaSiO_4_:*x*Eu^2+^/Eu^3+^ phosphors as a function of doping content.

The optical performance of the resultant phosphors was investigated by measuring their emission and excitation spectra. Figure 2a depicts the excitation spectra of the Li_2_CaSiO_4_:0.03Eu^2+^/Eu^3+^ phosphors monitored at 480 and 702 nm. As displayed, when the monitoring wavelength was 480 nm, the excitation spectrum consisted of two broad bands arising from the 4f.-5d transition of Eu^2+^ ions^30,31^. In comparison, when the monitoring wavelength was switched to 702 nm, only several sharp peaks were seen in the excitation spectrum and the broad intense bands vanished. Specially, these narrow peaks located at 319, 362, 376, 384, 395 and 416 nm pertained to the intra-4f transitions of Eu^3+^ ions from the ^7^F_0_ level to ^5^H_6_, ^5^D_4_, ^5^G_2_, ^5^G_3_, ^5^L_6_ and ^5^D_3_ levels, respectively^32,33^. Note that, these two excitation spectra exhibited an overlap at the wavelength of 395 nm (see Fig. 2a). Thus, to allow the studied samples present superior optical performance, we selected it as the excitation wavelength. The emission spectrum of the Li_2_CaSiO_4_:0.03Eu^2+^/Eu^3+^ phosphors excited by 395 nm is illustrated in Fig. 2b. Evidently, the emission profile was composed of an intense broad band and four sharp peaks. Among them, the intense broad band centered at 480 nm was attributed to the featured emissions of Eu^2+^ ions, whereas these narrow peaks located at 594 (^5^D_0_ → ^7^F_1_), 617 (^5^D_0_ → ^7^F_2_), 654 (^5^D_0_ → ^7^F_3_) and 702 (^5^D_0_ → ^7^F_4_) nm all pertained to the characteristic emissions of Eu^3+^ ions^33,34^. The simultaneous observation of the featured emissions of Eu^3+^ and Eu^2+^ ions in the prepared samples further uncovered that the Eu^3+^ ions were partially transferred to the Eu^2+^ ions. The energy level diagram of Eu^3+^ and Eu^2+^ ions was constructed and shown in Fig. 2c so as to describe the near-ultraviolet light triggered the visible emission mechanism in the Li_2_CaSiO_4_:*x*Eu^2+^/Eu^3+^ system.

It has been verified that the luminescent properties of the rare-earth ions based materials were sensitivity to the doping content. For the aim of digging out the optimal doping concentration, the concentration dependent photoluminescent properties of the Li_2_CaSiO_4_:*x*Eu^2+^/Eu^3+^ phosphors excited by 395 nm was studied and the corresponding results are presented in Fig. 2d. It is shown in Fig. 2d that the emission profiles did not vary with the addition of Eu^2+^/Eu^3+^ ions, whereas the emission intensities of Eu^2+^ and Eu^3+^ ions were determined to be relied on the dopant content. In particular, the emission intensities of Eu^2+^ and Eu^3+^ ions were all elevated with the increment of the doping concentration and their maximum values were gained when *x* = 0.03. However, the quenched emission intensity, which was caused by the concentration quenching effect, arose when the dopant content was over 3 mol%, as described in Fig. 2f. The colorific behaviors of the resultant phosphors were investigated and their CIE coordinates, which were estimated from the emission spectra, are presented in Fig. 2g and Table S1. Significantly, with changing the doping content in the range of 0.5–4 mol%, we found that the emitting color of the resultant compounds was varied and their CIE coordinates were changed from (0.394,0.326) to (0.293,0.274) (see Fig. 2g and Table S1). It is shown in Fig. S4 that FIR values of the Eu^2+^ to Eu^3+^ ions in the designed samples varied from each other and it can be responsible for the obtained multicolor emissions.

The room temperature decay curves of the Li_2_CaSiO_4_:*x*Eu^2+^/Eu^3+^ phosphors with the doping concentration of 3 mol% excited at 395 nm and monitored at different wavelengths of 480 and 702 nm were tested, as illustrated in Fig. S5a,b, respectively. As presented in Fig. S5a, the decay curve of Eu^2+^ ions (λ_ex_ = 395 nm, λ_em_ = 480 nm) was able to be fitted through utilizing a second-order exponential decay mode, which may be assigned to the nonradiative energy transfer process involving Eu^2+^ ions and defects^21^, as described below:

4 $$\mathrm{I}\left(t\right)={I}_{0}+{A}_{1}exp\left(-t/{\tau }_{1}\right)+{A}_{2}exp\left(-t/{\tau }_{2}\right),$$

In this equation, *I*_0_ and *I*(*t*) denote the emission intensities at time *t* = 0 and *t*, respectively, *A*_*i*_ (*i* = 1, 2) is constant, *τ*_1_ and *τ*_2_ are attributed to the decay time for exponential components, respectively. Besides, the following function can be applied to estimate the average lifetime (i.e., *τ*_*avg*_):

5 $${\tau }_{avg}=\frac{{A}_{1}{\tau }_{1}^{2}+{A}_{2}{\tau }_{2}^{2}}{{A}_{1}{\tau }_{1}+{A}_{2}{\tau }_{2}},$$

Consequently, the lifetime of the Eu^2+^ ions was found to be around 11.46 μs. In comparison, the decay curve of the Eu^3+^ ions (λ_ex_ = 395 nm, λ_em_ = 702 nm) can be fitted by employing a single exponential decay mode (see Fig. S5b), as demonstrated below:

6 $$I\left(t\right)={I}_{0}+Aexp\left(-t/\tau \right)$$

where *I*_0_ and *I*(*t*) denote the emission intensities at time *t* = 0 and *t*, respectively, *A* refers to the coefficient, *τ* is related to the decay time. Obviously, the decay time of the Eu^3+^ ions in the studied samples was 1898.68 μs.

**Figure 3:**
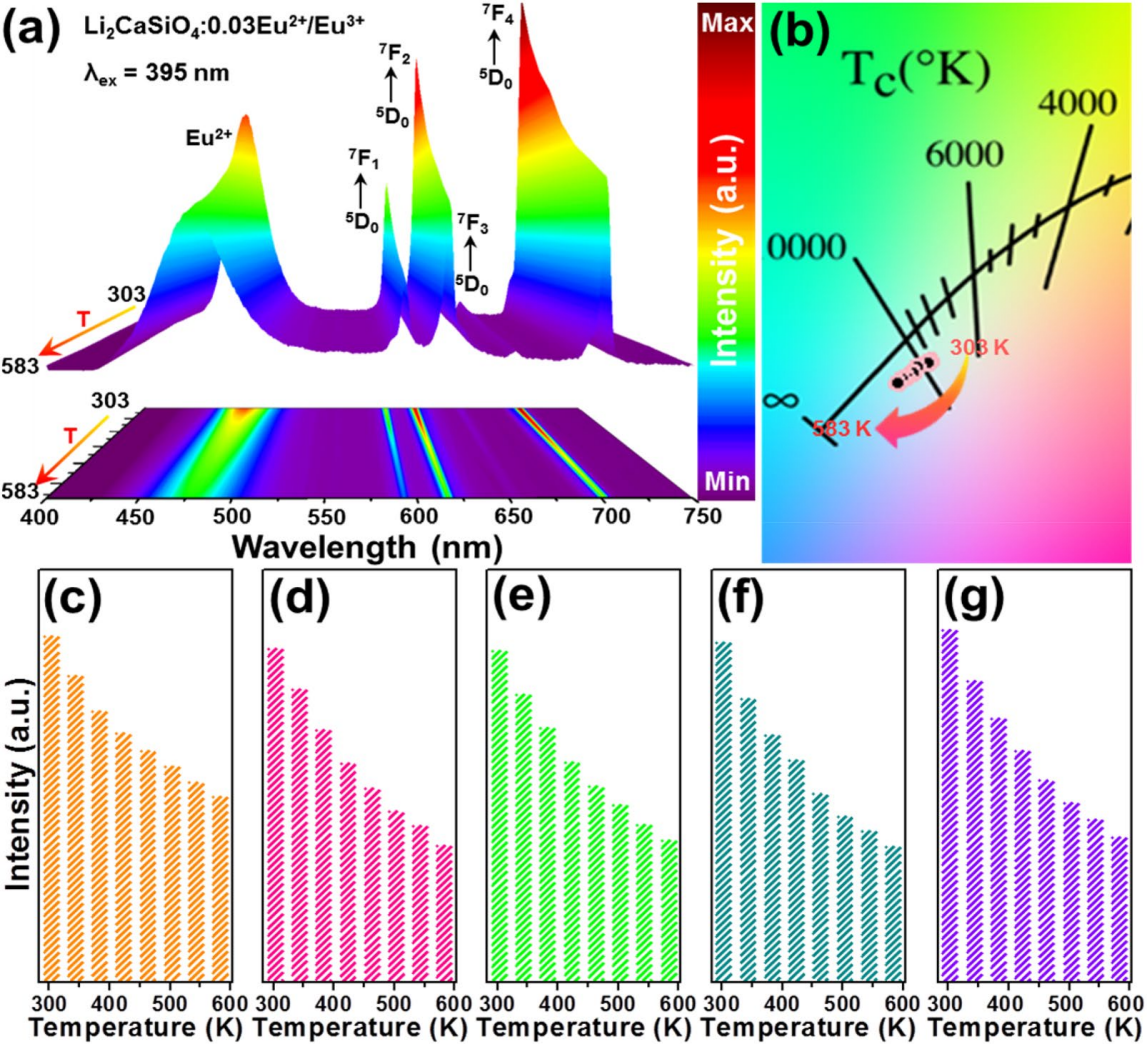
(**a**) Temperature-dependent emission spectra of the Li_2_CaSiO_4_:0.03Eu^2+^/Eu^3+^ phosphors excited by 395 nm. (**b**) CIE coordinate diagram of the Li_2_CaSiO_4_:0.03Eu^2+^/Eu^3+^ phosphors as a function of temperature. Emission intensities of (**c**) Eu^2+^ ions, (**d**) ^5^D_0_ → ^7^F_1_ transition, (**e**) ^5^D_0_ → ^7^F_2_ transition, (**f**) ^5^D_0_ → ^7^F_4_ transition and (**g**) total ^5^D_0_ → ^7^F_J_ (J = 1, 2 and 4) transitions.

For purpose of exploring the feasibility of the Eu^2+^/Eu^3+^-coactivated Li_2_CaSiO_4_ phosphors for contactless optical thermometry, their temperature-dependent emission spectra in the range of 303–583 K were examined. Figure 3a illustrates the temperature-dependent emission spectra of the Li_2_CaSiO_4_:0.03Eu^2+^/Eu^3+^ phosphors excited by 395 nm. It is significant that the emission profiles were not affected by the temperature, while the emission intensities of the Eu^2+^ and Eu^3+^ ions were dependent on temperature. Moreover, the temperature-dependent color coordinates of the studied samples, which were evaluated from the measured emission spectra, are listed in Table S2. Obviously, the CIE coordinates were sensitive to the temperature and their values were shifted from (0.293, 0.274) to (0.276, 0.260) when the surrounding temperature was changed in the range of 303–583 K, resulting in the tunable emissions at high temperature (see Fig. 3b). Furthermore, the temperature-dependent integrated emission intensities of Eu^2+^ ions, 594 nm (^5^D_0_ → ^7^F_1_) transition, 617 nm (^5^D_0_ → ^7^F_2_), 654 nm (^5^D_0_ → ^7^F_4_) and total ^5^D_0_ → ^7^F_J_ (J = 1, 2 and 4) transitions of Eu^3+^ ions were investigated and demonstrated in Fig. 3c–g, respectively. As disclosed, although all the emission intensities decreased, which was induced by the thermal quenching effect, with rising the temperature in the range of 303–583 K, the emission intensities of Eu^2+^ and Eu^3+^ ions exhibited diverse decline rates. Consequently, the accurate optical temperature measurement is able to be realized through analyzing the temperature-dependent FIR value of the emission intensities between the Eu^2+^ and Eu^3+^ ions.

**Figure 4:**
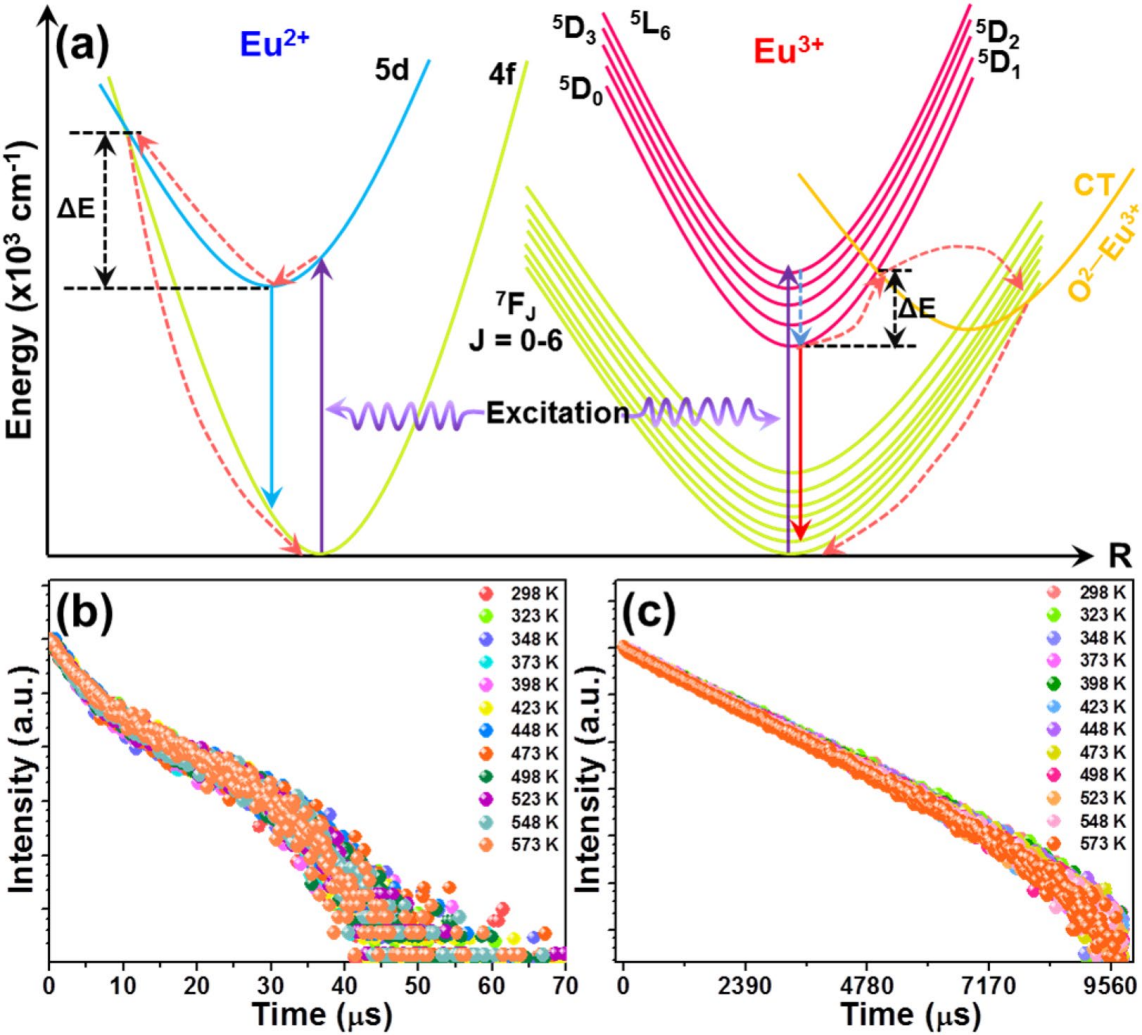
(**a**) Thermal quenching mechanism of the Eu^2+^ and Eu^3+^ ions in the Li_2_CaSiO_4_:*x*Eu^2+^/Eu^3+^ phosphors. Temperature-dependent decay curves of (**b**) Eu^2+^ (λ_ex_ = 395 nm, λ_em_ = 480 nm) and (**c**) Eu^3+^ (λ_ex_ = 395 nm, λ_em_ = 702 nm) ions in the Li_2_CaSiO_4_:0.03Eu^2+^/Eu^3+^ phosphors.

As pointed out above, the thermal quenching effect occurred at elevated temperature, resulting in the declined emission intensities of Eu^2+^ and Eu^3+^ ions. In order to explain thermal quenching mechanism as well as clarify the origination of the different temperature-dependent emission intensities of Eu^2+^ and Eu^3+^ ions, the schematic configurational coordinate diagram of Eu^2+^ and Eu^3+^ ions was constructed and presented in Fig. 4a. As for the Eu^2+^ ions, the crossover relaxation between the excited level of 5d and ground state of 4f can contribute to the quenched emission intensity at elevated temperature. Specially, with rising the temperature, electrons populated at the bottom of the excited level would shift to the intersection between the parabolas of the 4f ground state and the 5d excited level (see Fig. 4a). After that, these populated electrons will nonradiatively decay to the ground state, leading to the declined emission intensity at high temperature. On the other hand, unlike the Eu^2+^ ions, the Eu^3+^ ions show totally different thermal quenching mode since its excited levels and ground states do not have any crossover points^20^. As presented, electrons can be pumped from the ground state to the ^5^L_6_ excited level excited at 395 nm, and then the nonradiative transition occurs, leading to the formation of ^5^D_0_ level. Subsequently, the emissions originating from the ^5^D_0_ to ^7^F_J_ (J = 1, 2, 3, 4) levels appeared. Note that, the charge transfer (CT) band of O^2−^ → Eu^3+^ is located at a relatively low energy which makes it possible to supply a pathway for the electrons located at the ^5^D_0_ level to nonradiatively return to the ground states^20,35^. Therefore, the thermal quenching mode of Eu^3+^ ions is the thermal activation of the electrons from the ^5^D_0_ excited level to the CT band of O^2−^ → Eu^3+^, as demonstrated in Fig. 4a. Finally, these generated electrons will return to the ground state through a nonradiative pathway and the quenched emission intensities are observed at elevated temperature. In order to confirm the aforementioned guess, the temperature-dependent decay curves of the Eu^2+^ and Eu^3+^ ions were examined, as shown in Fig. 4b,c, respectively. As is known to all, the thermal quenching effect can be realized by three diverse pathways of energy transfer from the luminescent states, cascade multiphonon relaxation and crossover process at high temperature^36,37^. Notably, these three different channels are able to be discriminated by utilizing the temperature-dependent lifetime. It is shown in Fig. 4b,c that the decay carves of the Eu^2+^ and Eu^3+^ ions hardly changed with the increment of temperature, indicating that the thermal quenching effect can not be contributed by the cascade multiphonon relaxation. Additionally, we also found that the both the decay time of the Eu^2+^ and Eu^3+^ ions were insensitive to the temperature (see Fig. 4b,c), implying that the energy transfer from the luminescent levels was not the potential route for the thermal quenching effect. As a consequence, it is reasonable for us to conclude that the thermal quenching mechanisms of the Eu^2+^ and Eu^3+^ ions in the Li_2_CaSiO_4_ host were all dominated by the crossover relation process.

**Figure 5:**
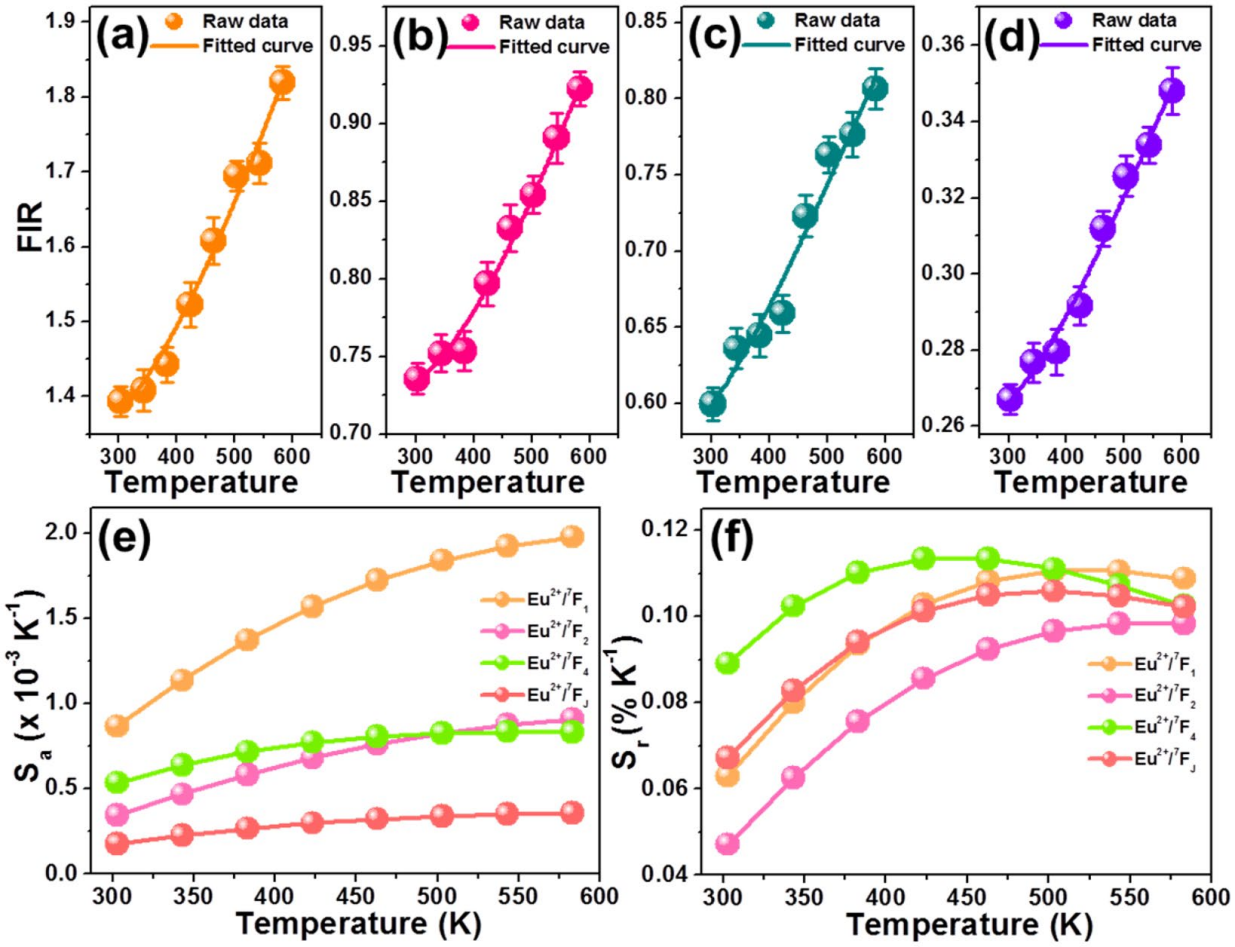
FIR values of the (**a**) Eu^2+^/^7^F_1_, (**b**) Eu^2+^/^7^F_2_, (**c**) Eu^2+^/^7^F_4_ and (**d**) total Eu^2+^/^7^F_J_ in the Li_2_CaSiO_4_:0.03Eu^2+^/Eu^3+^ phosphors as a function of temperature. Dependence of (**e**) *S*_*a*_ and (**f**) *S*_*r*_ value on the temperature for the Li_2_CaSiO_4_:0.03Eu^2+^/Eu^3+^ phosphors.

Based on the recorded emission spectra shown in Fig. 3a, the temperature-dependent FIR values of Eu^2+^ ions to ^5^D_0_ → ^7^F_1_ transition (i.e., Eu^2+^/^7^F_1_), Eu^2+^ ions to ^5^D_0_ → ^7^F_2_ transition (i.e., Eu^2+^/^7^F_2_), Eu^2+^ ions to ^5^D_0_ → ^7^F_4_ transition (i.e., Eu^2+^/^7^F_4_) and Eu^2+^ ions to ^5^D_0_ → ^7^F_J_ (J = 1, 2, 4) transition (i.e., total Eu^2+^/^7^F_J_) were evaluated and the corresponding results are depicted in Fig. 5a–d, respectively. Significantly, with elevating the temperature in the range of 303–583 K, all of the calculated FIR values increased gradually. Nevertheless, these calculated FIR values based on the different emission combinations exhibited various values which indicated that the sensitivities of the studied samples may be tuned through choosing diverse emission combination. From previously reports, it is clear that the relation between the FIR value of the non-thermally coupled levels and temperature satisfies the following function^38,39^:

7 $$\mathrm{FIR}=Aexp\left(-B/T\right)+C$$

In this expression, the parameters of *A*,* B* and *C* are all constants. With the help of Eq. (7), these obtained temperature-dependent FIR values were fitted well, as shown in Fig. 5a–d. Particularly, the FIR expression of the Eu^2+^/^7^F_1_, Eu^2+^/^7^F_2_, Eu^2+^/^7^F_4_ and total Eu^2+^/^7^F_J_ combinations were determined to be FIR = 5.02 exp (− 1345.98/T) + 1.32, FIR = 2.53 exp (− 1437.86/T) + 0.71, FIR = 1.71 exp (− 1105.18/T) + 0.56, FIR = 0.84 exp (− 1261.18/T) + 0.25, respectively. To get deeper insight into the optical thermometry properties of the luminescent materials, the temperature-dependent *S*_*a*_ and *S*_*r*_ values should be investigated since they can intuitively reflect the temperature sensing ability of the luminescent materials. Through utilizing the following functions, the *S*_*a*_ and *S*_*r*_ values are available to be achieved, as defined below^40,41^:

8 $${S}_{a}=\frac{d\mathrm{FIR}}{dT}=Aexp\left(-B/T\right)\times \left(B/{T}^{2}\right),$$

9 $${S}_{r}=\frac{1}{\mathrm{FIR}}\frac{d\mathrm{FIR}}{dT}\times 100\%=\frac{Aexp\left(B/T\right)}{Aexp\left(-B/T+C\right)}\times \frac{B}{{T}^{2}}\times100\%,$$

where the values of *A*, *B* and *C* were the same as presented in Eq. (7). On the basis of Eqs. (8) and (9) along with the fitted values shown in Fig. 5a–d, the *S*_*a*_ and *S*_*r*_ values of the Li_2_CaSiO_4_:0.03Eu^2+^/Eu^3+^ phosphors as a function of temperature were achieved, as demonstrated in Fig. 5e,f, respectively. As disclosed in Fig. 5e, the *S*_*a*_ values showed an upward tendency with the temperature, reaching their maximum values when the temperature was 583 K. Specially, for the combinations of the Eu^2+^/^7^F_1_, Eu^2+^/^7^F_2_, Eu^2+^/^7^F_4_ and total Eu^2+^/^7^F_J_, their maximum *S*_*a*_ values were around 0.0020 K^−1^, 0.0009 K^−1^, 0.0008 K^−1^ and 0.0003 K^−1^, respectively. Furthermore, it is shown in Fig. 5f that the *S*_*r*_ values firstly increased with the temperature, achieving their maximum values, and then they started to decease with further increasing the temperature. The maximum *S*_*r*_ values of the Eu^2+^/^7^F_1_, Eu^2+^/^7^F_2_, Eu^2+^/^7^F_4_ and Eu^2+^/^7^F_J_ combinations were 0.110% K^−1^, 0.098% K^−1^, 0.113% K^−1^ and 0.105% K^−1^, respectively. Evidently, the *S*_*a*_ and *S*_*r*_ values of the studied samples can be facilely modulated through utilizing diverse emission combinations (i.e., spatial mode).

The reversibility and thermal stability of the studied samples were investigated through analyzing the temperature-caused switching of FIR values in the range of 303–583 K. Figure S6 shows the temperature-caused switching of FIR values of the Li_2_CaSiO_4_:0.03Eu^2+^/Eu^3+^ phosphors. It is clear that the FIR values were reversible and repeatable even after six heating–cooling processes. Note that, the phase structure of the resultant compounds also did not change after six heating–cooling processes, as shown in Fig. S7. These results implied that the resultant compounds had good reversibility and stability. As disclosed in previous reports, the temperature uncertainty of the optical temperature sensor based on the luminescent compounds is able to be determined by means of following functions^42–44^:

10 $$\frac{\delta FIR}{FIR}=\sqrt{{\left(\frac{\delta {I}_{1}}{{I}_{1}}\right)}^{2}+{\left(\frac{\delta {I}_{2}}{{I}_{2}}\right)}^{2}}$$

11 $$\updelta T=\frac{1}{{S}_{r}}\times \frac{\delta FIR}{FIR}$$

where *I*_1_ refers to the emission intensity of Eu^2+^ ions, *I*_2_ stands for the emission intensities of the Eu^3+^ ions originating from the ^5^D_0_ → ^7^F_J_ transitions, δ*I*_1_ and δ*I*_2_ are ascribed to the errors of *I*_1_ and *I*_2_, respectively, and δ*T* is the temperature uncertainty. Via these above expressions, the δ*T* values were estimated to be 0.139–0.248 K (303–583 K), 0.146–0.352 K (303–583 K), 0.116–0.433 K (303–583 K), 0.102–0.383 K (303–583 K), respectively, when the combinations of Eu^2+^/^7^F_1_, Eu^2+^/^7^F_2_, Eu^2+^/^7^F_4_ and Eu^2+^/^7^F_J_ were employed.

**Figure 6:**
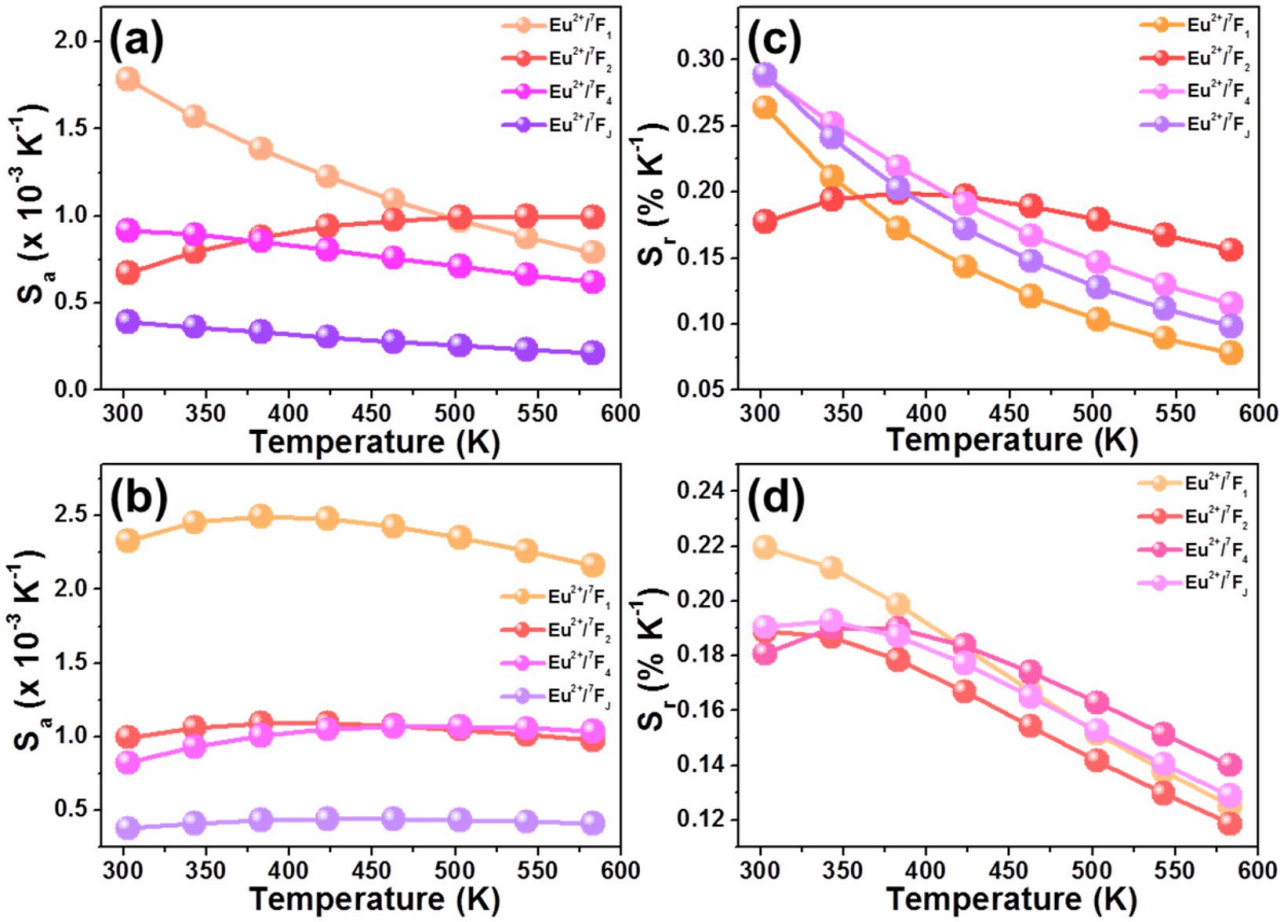
*S*_*a*_ value of the (**a**) Li_2_CaSiO_4_:0.005Eu^2+^/Eu^3+^ and (**b**) Li_2_CaSiO_4_:0.04Eu^2+^/Eu^3+^ phosphors at different temperature. *S*_*r*_ value of the (**c**) Li_2_CaSiO_4_:0.005Eu^2+^/Eu^3+^ and (**d**) Li_2_CaSiO_4_:0.04Eu^2+^/Eu^3+^ phosphors as a function of temperature.

From the room temperature emission spectra (Fig. 2d), one knows that the relative emission intensities of the Eu^2+^ and Eu^3+^ ions were impacted by the doping content. Besides, the room temperature FIR values of Eu^2+^ to Eu^3+^ ions were dependent on the doping concentration (Fig. S4), suggesting that the sensitivities of the studied samples may be affected by the dopant content. For the sake of verifying this speculation, the thermometric properties of the Li_2_CaSiO_4_:*x*Eu^2+^/Eu^3+^ phosphors with different doping contents of 0.5 and 4 mol% were explored. Upon the irradiation of 395 nm, the emission spectra of the Li_2_CaSiO_4_:0.005Eu^2+^/Eu^3+^ and Li_2_CaSiO_4_:0.04Eu^2+^/Eu^3+^ phosphors as a function of temperature in the range of 303–583 K were measured, as illustrated in Fig. S8a,b, respectively. It can be seen that the emission profiles of these two compounds varied little with boosting the temperature, whereas the emission intensities of the Eu^2+^ and Eu^3+^ ions were affected by the temperature. Similar as those of in the resultant phosphors with optimum doping content, the emission intensities of the Eu^2+^ ions, 594 nm (^5^D_0_ → ^7^F_1_) transition, 617 nm (^5^D_0_ → ^7^F_2_), 654 nm (^5^D_0_ → ^7^F_4_) and total ^5^D_0_ → ^7^F_J_ (J = 1, 2 and 4) transitions of Eu^3+^ ions in the Li_2_CaSiO_4_:0.005Eu^2+^/Eu^3+^ and Li_2_CaSiO_4_:0.04Eu^2+^/Eu^3+^ phosphors all decreased monotonously with the temperature (see Fig. S9). Nevertheless, these emissions exhibited various decreasing rates which make them were suitable for contactless optical thermometry. Figure S10 presents the FIR values of these two compounds as a function of temperature. Clearly, the whole FIR values increased gradually with the temperature and they can be perfectly fitted by Eq. (7), as shown in Fig. S8. Based on the fitted results as well as Eqs. (8) and (9), the temperature-dependent *S*_*a*_ and *S*_*r*_ values of the Li_2_CaSiO_4_:0.005Eu^2+^/Eu^3+^ and Li_2_CaSiO_4_:0.04Eu^2+^/Eu^3+^ phosphors were calculated and their corresponding results are presented in Fig. 6. As uncovered, both the *S*_*a*_ and *S*_*r*_ values of these two compounds were sensitive to the emission combinations of Eu^2+^/Eu^3+^ ions which further confirmed that the temperature sensing ability of the synthesized phosphors can be manipulated through adjusting the spatial mode. As shown in Fig. 6a,b, the maximum *S*_*a*_ values of the Li_2_CaSiO_4_:0.005Eu^2+^/Eu^3+^ and Li_2_CaSiO_4_:0.04Eu^2+^/Eu^3+^ phosphors were revealed to be about 0.0018 K^−1^ and 0.0025 K^−1^, respectively, at 303 K when the Eu^2+^/^7^F_1_ combination was used. Furthermore, the maximum *S*_*r*_ value of the Li_2_CaSiO_4_:0.005Eu^2+^/Eu^3+^ phosphors was 0.289% K^−1^ when the combination of total Eu^2+^/^7^F_J_ was employed (see Fig. 6c), whereas that of the Li_2_CaSiO_4_:0.04Eu^2+^/Eu^3+^ phosphors reached up to 0.219% K^−1^ when the Eu^2+^/^7^F_J_ mode was adopted (see Fig. 6d). According to these aforementioned results, it is obvious that the *S*_*a*_ and *S*_*r*_ values of the prepared phosphors were totally different which were greatly impacted by the doping concentration (see Table 1), implying that the optical thermometric performance of the Eu^2+^/Eu^3+^-coactivated Li_2_CaSiO_4_ phosphors was able to modified by adjusting the doping content aside from selecting different spatial mode. Additionally, compared previously reported optical thermometers based on rare-earth activated compounds, the designed Li_2_CaSiO_4_:*x*Eu^2+^/Eu^3+^ phosphors showed relatively good thermometric behaviors (see Table 1), proving that the resultant phosphors were promising luminescent materials for contactless optical thermometers.

**Table 1:** Temperature range, excitation wavelength, maximum *S*_*a*_ and *S*_*r*_ values of the rare-earth ions based optical thermometers.

Compounds	Temperature (K)	λ_ex_ (nm)	*S*_*a*_ (K^−1^)	*S*_*r*_ (K^−1^)	Reference
YVO_4_:Nd^3+^/Yb^3+^	123–420	320	–	0.25%	^8^
LaOBr:Ce^3+^/Tb^3+^	293–433	350	–	0.42%	^12^
BaTiO_3_:Er^3+^	300–450	380	0.0032	–	^45^
YF_3_:Eu^3+^/Tb^3+^	303–563	377	0.0013	–	^46^
Y_2_O_3_:Tm^3+^/Yb^3+^	293–553	80	0.0028	0.66%	^47^
Ba_5_Gd_8_Zn_4_O_21_:Er^3+^/Yb^3+^	200–490	980	0.0032	–	^48^
Li_2_CaSiO_4_:0.005Eu^2+^/Eu^3+^	303–583	395	0.0018	0.289%	This work
Li_2_CaSiO_4_:0.03Eu^2+^/Eu^3+^	303–583	395	0.0020	0.113%	This work
Li_2_CaSiO_4_:0.04Eu^2+^/Eu^3+^	303–583	395	0.0025	0.219%	This work

## Conclusions

In summary, through utilizing a simple solid-state reaction technique, the Eu^2+^/Eu^3+^-coactivated Li_2_CaSiO_4_ phosphors with multicolor emissions were prepared. Upon 395 nm irradiation, both the featured emissions of Eu^2+^ and Eu^3+^ ions were seen in the prepared phosphors and their maximum values were obtained when the doping content was 3 mol%. Based on the energy level diagram of Eu^2+^ and Eu^3+^ ions, the crossover relaxation was found to be responsible for the thermal quenching effect which was further proved by the temperature-dependent decay time. Through analyzing the different responses of the emissions of Eu^2+^ and Eu^3+^ ions, the temperature sensing ability of the designed compounds was investigated. It was revealed that the sensitivities of the Li_2_CaSiO_4_:*x*Eu^2+^/Eu^3+^ phosphors could be modulated through adjusting the spatial mode and doping concentration. When the Eu^2+^/^7^F_1_ combination was employed, the Li_2_CaSiO_4_:0.005Eu^2+^/Eu^3+^ phosphors exhibited a maximum *S*_*r*_ value of 0.289% K^−1^, while the Li_2_CaSiO_4_:0.04Eu^2+^/Eu^3+^ phosphors possessed a maximum *S*_*a*_ value of 0.0025 K^−1^. These achievements suggested that the Eu^2+^/Eu^3+^-coactivated Li_2_CaSiO_4_ phosphors may be promising candidates for contactless optical measurement. Ultimately, this work also proposed facile routes to modify the sensitivity of the rare-earth activated luminescent materials by means of tuning the spatial mode and doping content.

## Experimental section

### Materials and synthesis

The designed compounds with the general chemical formula of Li_2_Ca_1-*x*_SiO_4_:*x*Eu^2+^/Eu^3+^ (Li_2_CaSiO_4_:*x*Eu^2+^/Eu^3+^; where 0.005 ≤ *x* ≤ 0.04) were sintered by utilizing a simple high-temperature solid-state reaction technology. To carry out this experiment, the powders of Li_2_CO_3_, CaCO_3_, SiO_2_ and Eu_2_O_3_ with were purchased and used as the raw materials to synthesize these above compounds. On the basis of the designed stoichiometric proportion, the starting materials including Li_2_CO_3_, CaCO_3_, SiO_2_ and Eu_2_O_3_ were firstly weighted, and then thoroughly mixed by an agate mortar. Subsequently, these powders were kept in crucibles and heat at 900 °C for 5 h with the heating rate of 3 °C/min. After cooling down to the room temperature, the white powders were collected and ground. For the sake of making part of Eu^3+^ ions change to Eu^2+^ ions, the obtained white powders were heat at 600 °C for 20 min under a reducing atmosphere (N_2_:H_2_ = 95%:5%). Finally, the Li_2_CaSiO_4_:*x*Eu^2+^/Eu^3+^ powders were achieved and they can be used for further characterization.

### Materials characterization

The phase component, elemental compositions and morphological information of the final compounds was examined by means of an X-ray diffractometer with Cu Kα radiation (Bruker D8 Advance), PHI 5000 VersaProbe spectrometer and field-emission scanning electron microscope (FE-SEM; HITACHI SU3500) equipped with an energy dispersive X-ray (EDX) spectroscopy. The diffuse reflectance spectrum of the studied samples was recorded by utilizing the Cary 5000 UV–Vis spectrophotometer. The Edinburgh FS5 spectrofluorometer was adopted to detect the emission and excitation spectra of the prepared samples. The decay curves of the resultant phosphors were measured by utilizing a FLS920 fluorescence spectrophotometer. Through contacting a temperature control system (Linkam HFS600E-PB2), the emission spectra of the studied samples as a function of temperature were monitored by the Edinburgh FS5 spectrofluorometer.

## Supplementary information

Supplementary Information.
