# Supplementary material for: Facile modulation the sensitivity of Eu2+/Eu3+-coactivated Li2CaSiO4 phosphors through adjusting spatial mode and doping concentration
Source: Sci Rep. 2020 Nov 19;10:20180. doi: 10.1038/s41598-020-77185-w (PMC7678844; doi:10.1038/s41598-020-77185-w)
Supplement: Supplementary file 1 — Supplementary Information. [file 41598_2020_77185_MOESM1_ESM.docx]

**Facile modulation the sensitivity of Eu^2+^/Eu^3+^-coactivated Li_2_CaSiO_4_ phosphors through adjusting spatial mode and doping concentration**

**Luhui Zhou^a^, Peng Du^a*^and Li Li^b*^**

*^a^Department of Microelectronic Science and Engineering, School of Physical Science and Technology, Ningbo University, 315211 Ningbo, Zhejiang, China*

*^b^College of Science, Chongqing University of Posts and Telecommunications, Chongqing, China*

Corresponding authors: dupeng@nbu.edu.cn (P. Du); lilic@cqupt.edu.cn (L. Li)

**Table S1**. CIE coordinates of the Li_2_CaSiO_4_:*x*Eu^2+^/Eu^3+^ phosphors.

| Compounds | CIE coordinates | |
| --- | --- | --- |
|  | *x* | *y* |
| Li_2_CaSiO_4_:0.005Eu^2+^/Eu^3+^ | 0.394 | 0.326 |
| Li_2_CaSiO_4_:0.01Eu^2+^/Eu^3+^ | 0.384 | 0.303 |
| Li_2_CaSiO_4_:0.02Eu^2+^/Eu^3+^ | 0.360 | 0.289 |
| Li_2_CaSiO_4_:0.03Eu^2+^/Eu^3+^ | 0.293 | 0.274 |
| Li_2_CaSiO_4_:0.04Eu^2+^/Eu^3+^ | 0.328 | 0.276 |

**Table S2**. CIE coordinates of the Li_2_CaSiO_4_:0.03Eu^2+^/Eu^3+^ phosphors at diverse temperature.

| Temperature | CIE coordinates | |
| --- | --- | --- |
|  | *x* | *y* |
| 303 K | 0.293 | 0.274 |
| 343 K | 0.296 | 0.275 |
| 383 K | 0.294 | 0.273 |
| 423 K | 0.289 | 0.269 |
| 463 K | 0.285 | 0.267 |
| 503 K | 0.282 | 0.264 |
| 543 K | 0.278 | 0.262 |
| 583 K | 0.276 | 0.260 |


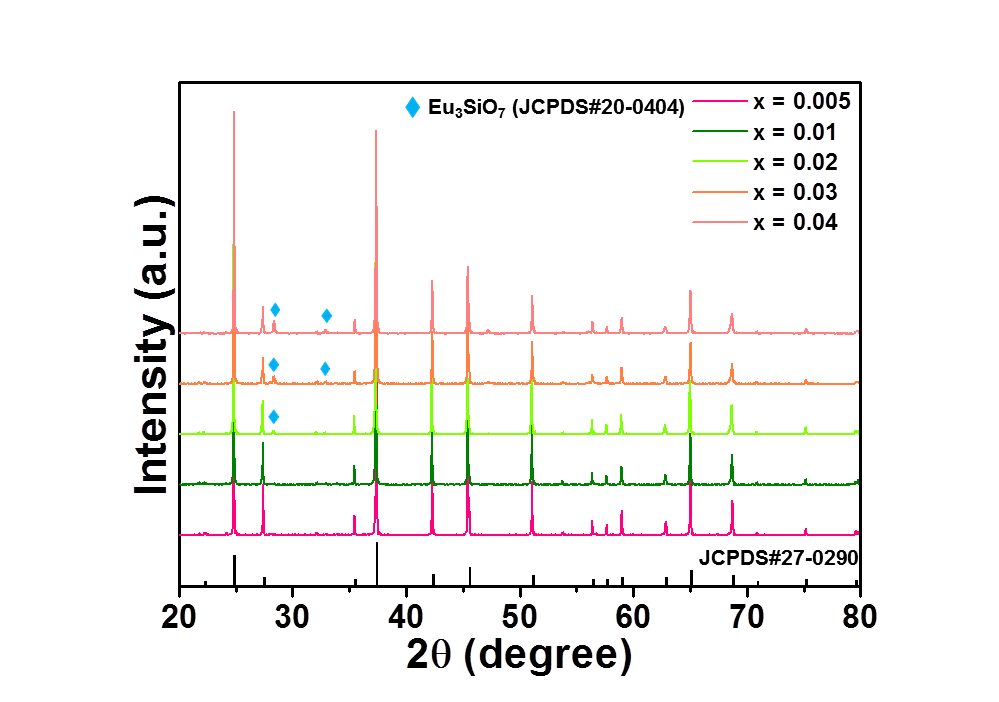


**Figure S1**. XRD patterns of the Li_2_CaSiO_4_:*x*Eu^2+^/Eu^3+^ phosphors.


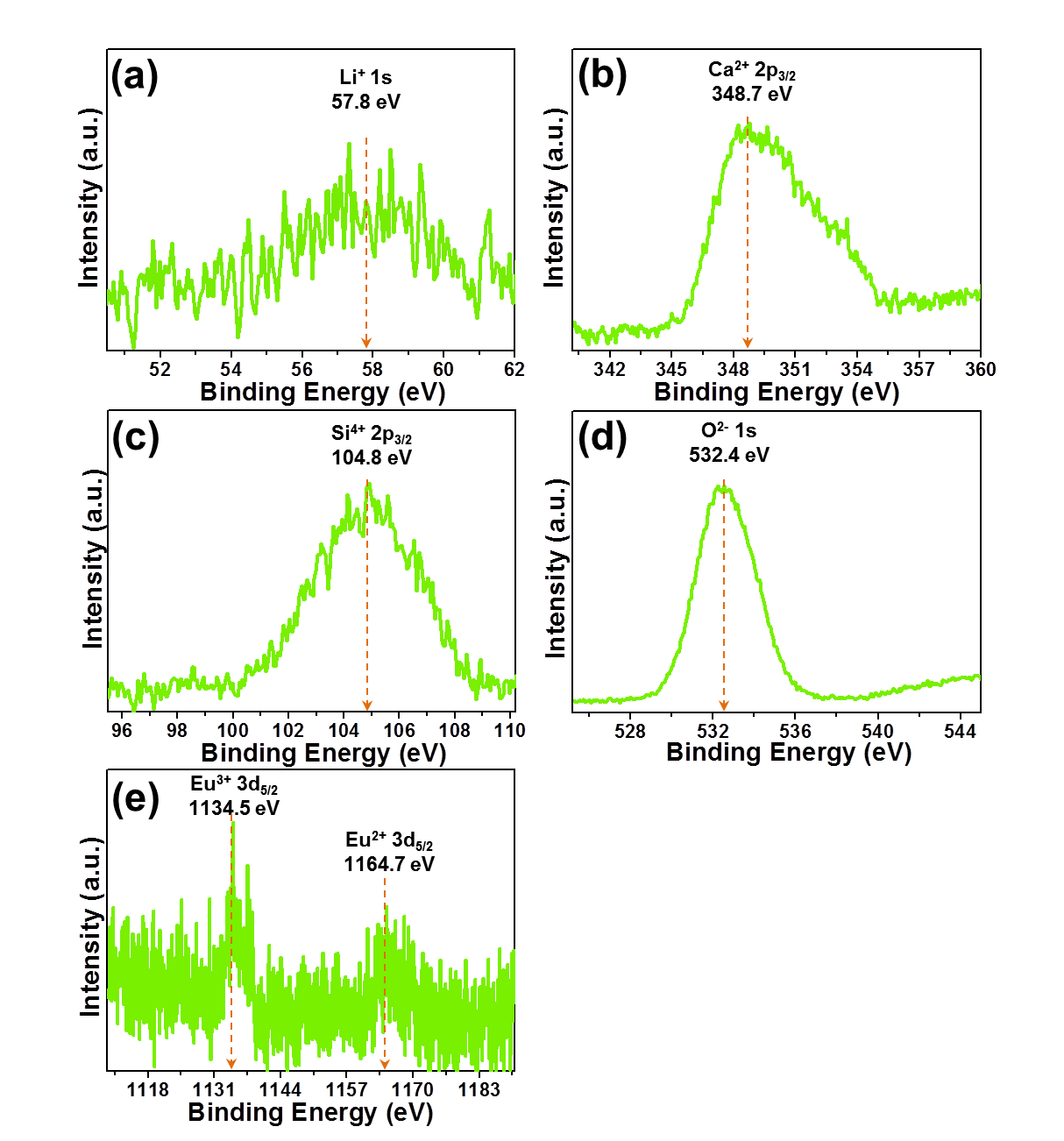


**Figure S2**. XPS spectra of (a) Li^+^ 1s, (b) Ca^2+^ 2p, (c) Si^4+^ 2p, (d) O^2-^ 1s and (e) Eu^3+^/Eu^2+^ 3d.


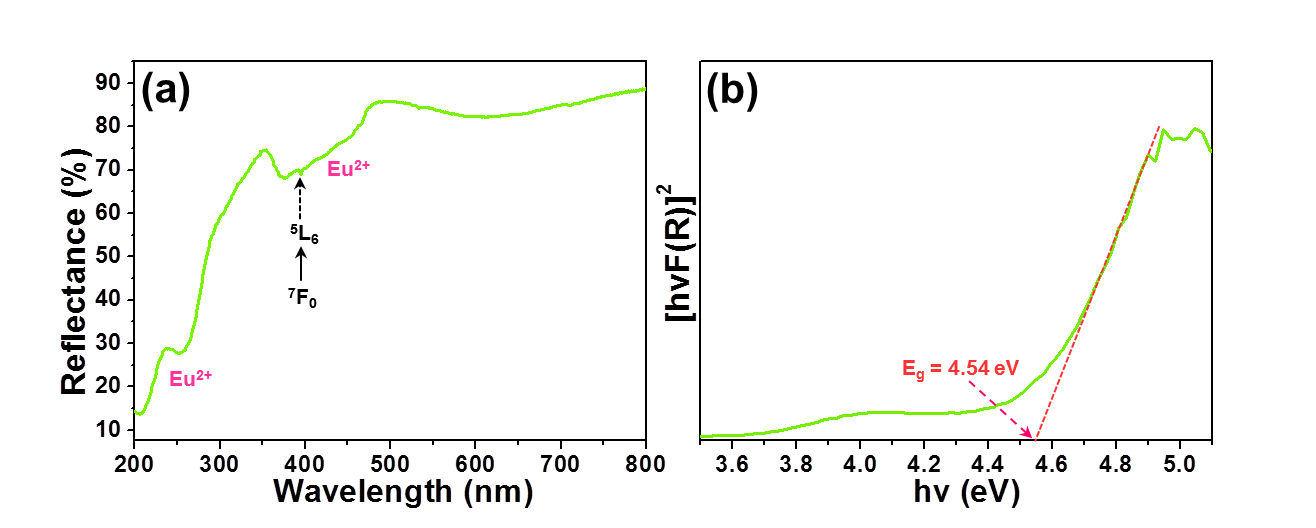


**Figure S3**. (a) Diffuse reflectance spectra of the Li_2_CaSiO_4_:0.03Eu^2+^/Eu^3+^ phosphors. (b) Calculation of the energy band gap of the Li_2_CaSiO_4_:0.03Eu^2+^/Eu^3+^ phosphors based on the Kubellka-Munk function.


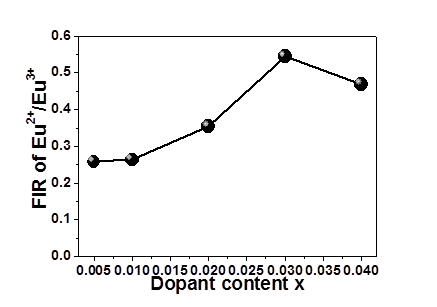


**Figure S4**. FIR values of Eu^2+^ to Eu^3+^ ions in the Li_2_CaSiO_4_:*x*Eu^2+^/Eu^3+^ phosphors as a function of doping concentration.


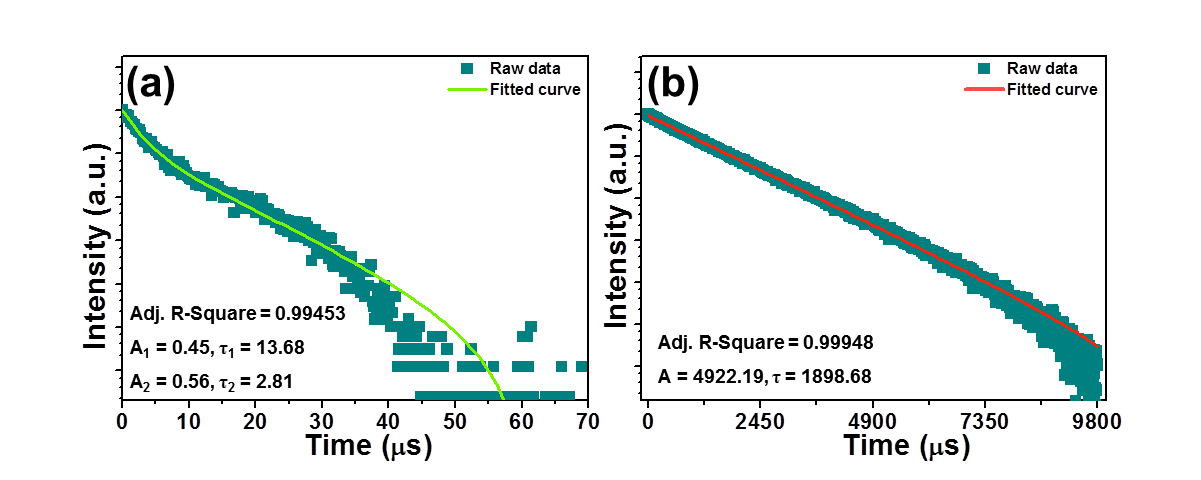


**Figure S5**. Decay curves of the (a) Eu^2+^ (λ_ex_ = 395 nm, λ_em_ = 480 nm) and (b) Eu^3+^ (λ_ex_ = 395 nm, λ_em_ = 702 nm) ions in the Li_2_CaSiO_4_:0.03Eu^2+^/Eu^3+^ phosphors at room temperature.


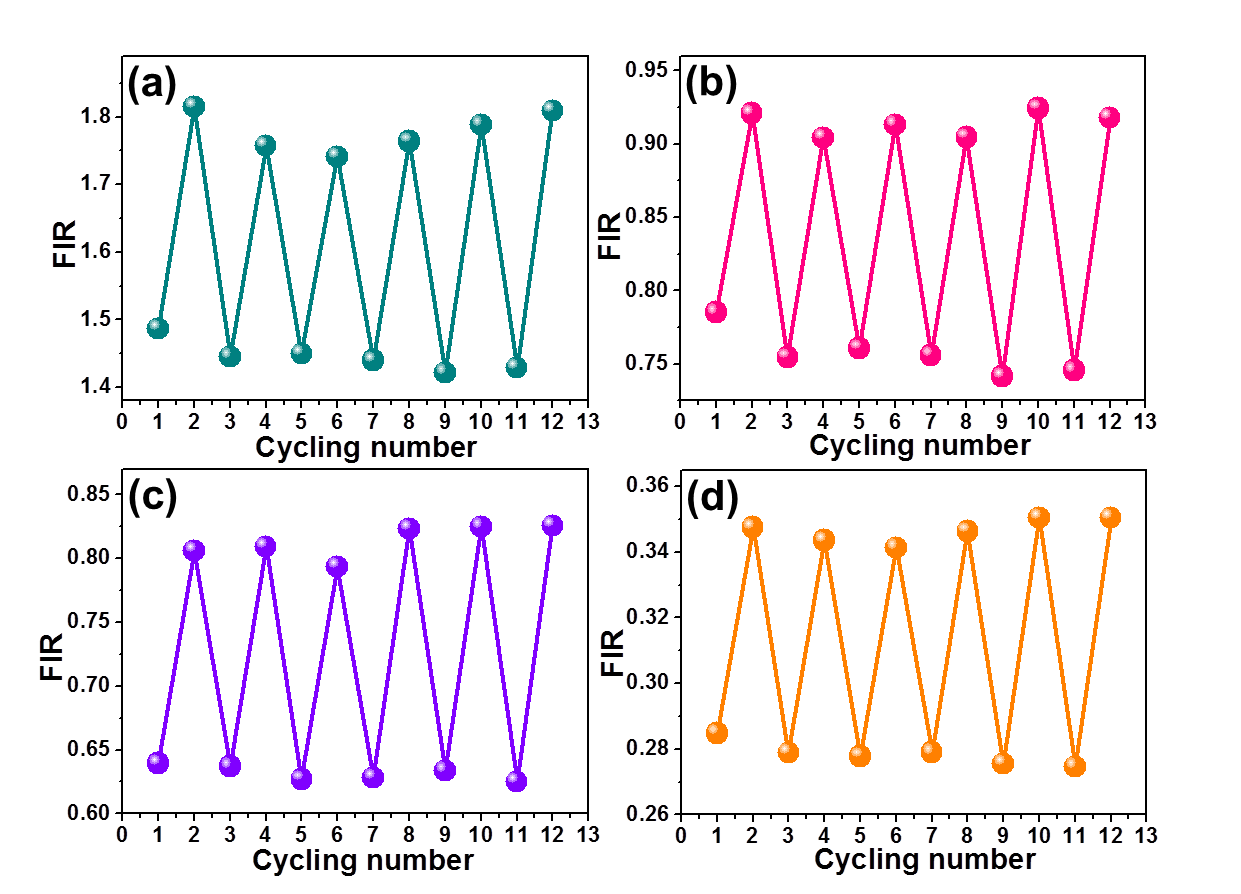


**Figure S6**. Temperature-induced switching of FIR values of the (a) Eu^2+^/^7^F_1_, (b) Eu^2+^/^7^F_2_, (c) Eu^2+^/^7^F_4_ and (d) total Eu^2+^/^7^F_J_ in the Li_2_CaSiO_4_:0.03Eu^2+^/Eu^3+^ phosphors in the range of 303-583 K.


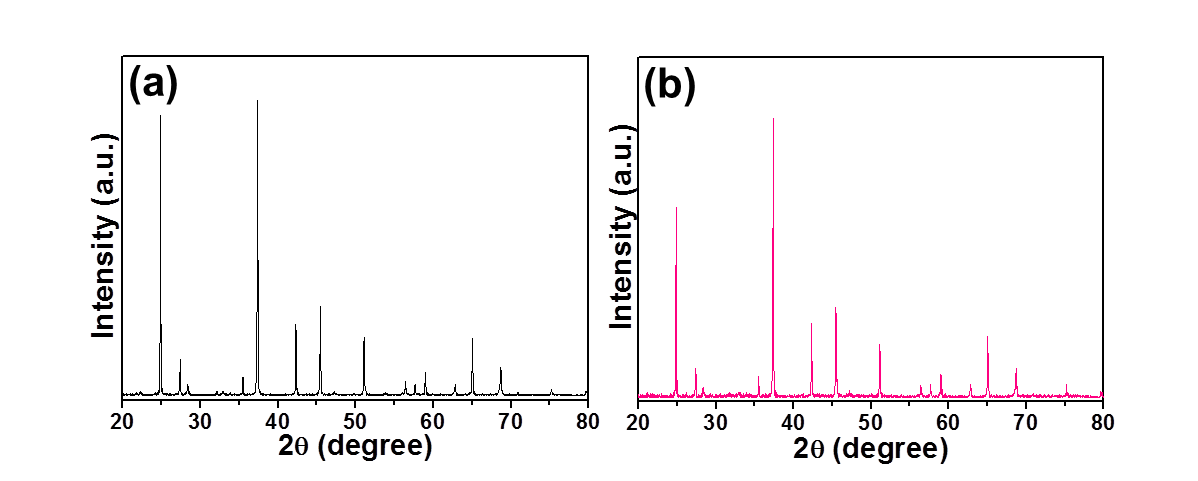


**Figure S7**. XRD patterns of Li_2_CaSiO_4_:0.03Eu^2+^/Eu^3+^ phosphors (a) without heating-cooling treatment and (b) with heating-cooling treatment.


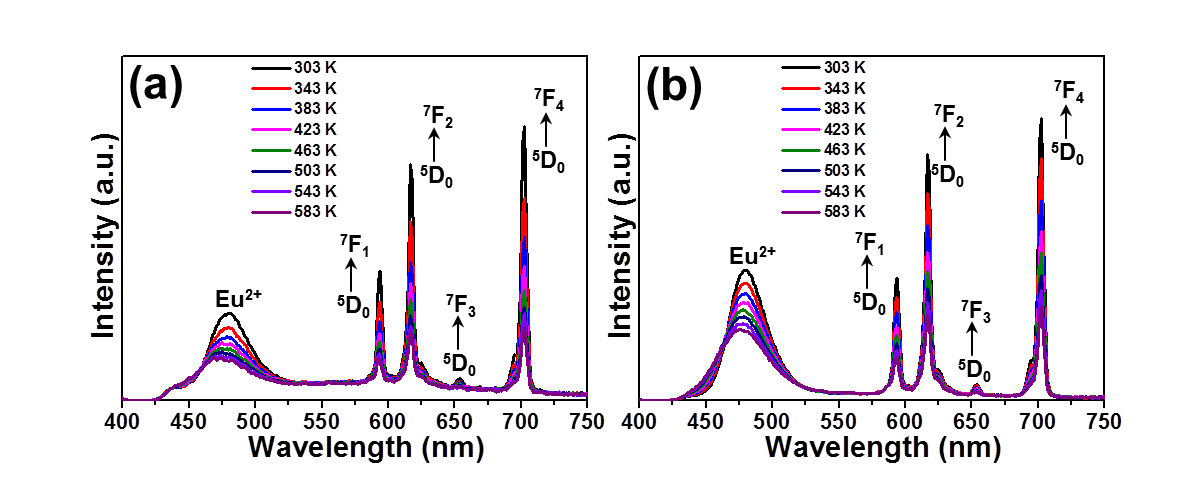


**Figure S8**. Temperature-dependent emission spectra of the Li_2_CaSiO_4_:0.005Eu^2+^/Eu^3+^ and (b) Li_2_CaSiO_4_:0.04Eu^2+^/Eu^3+^ phosphors excited by 395 nm.


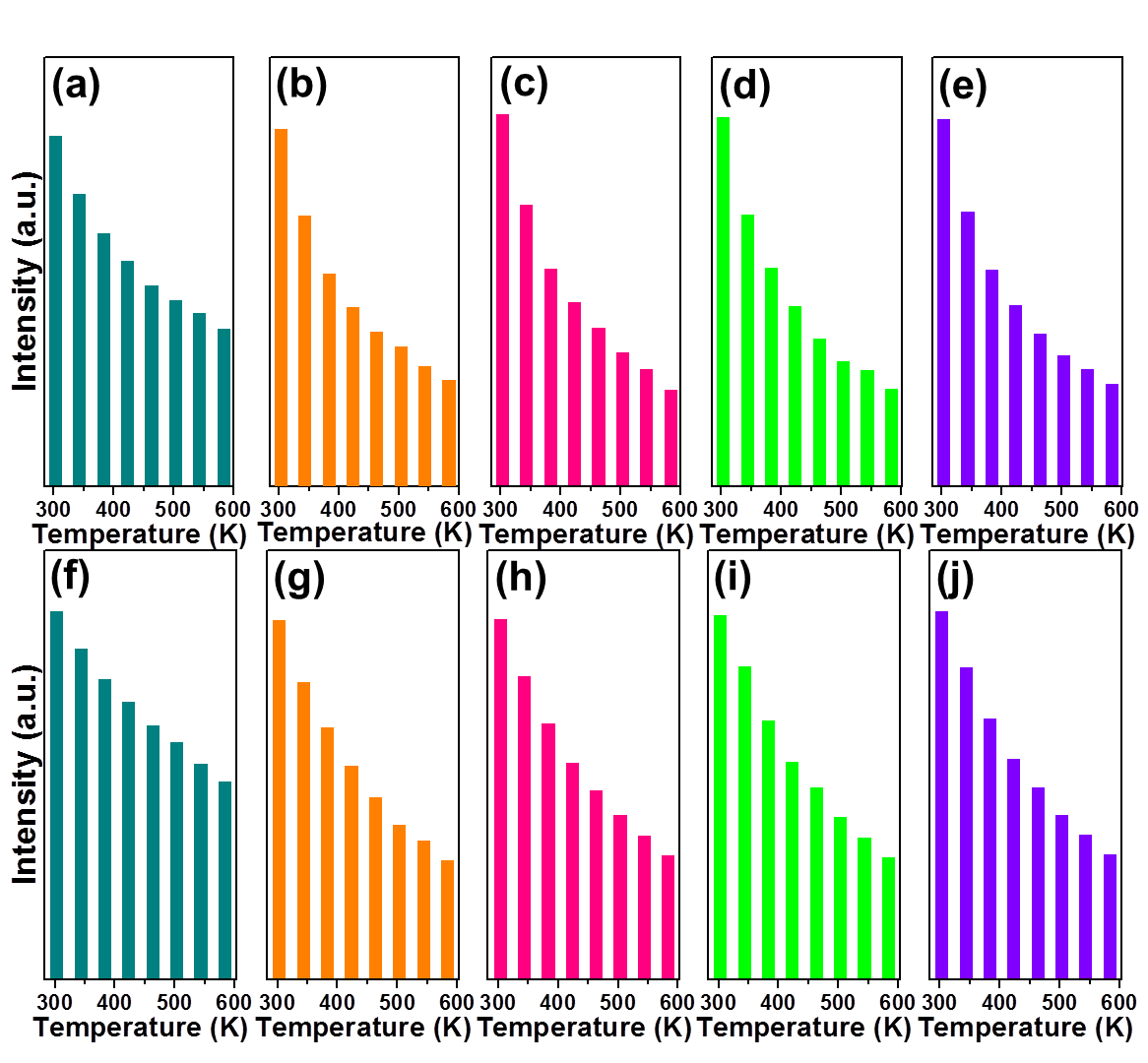


**Figure S9**. Emission intensities of (a) Eu^2+^ ions, (b) ^5^D_0_ → ^7^F_1_ transition, (c) ^5^D_0_ → ^7^F_2_ transition, (d) ^5^D_0_ → ^7^F_4_ transition and (e) total ^5^D_0_ → ^7^F_J_ (J = 1, 2 and 4) transitions in Li_2_CaSiO_4_:0.005Eu^2+^/Eu^3+^ phosphors. Emission intensities of (f) Eu^2+^ ions, (g) ^5^D_0_ → ^7^F_1_ transition, (h) ^5^D_0_ → ^7^F_2_ transition, (i) ^5^D_0_ → ^7^F_4_ transition and (j) total ^5^D_0_ → ^7^F_J_ (J = 1, 2 and 4) transitions in Li_2_CaSiO_4_:0.04Eu^2+^/Eu^3+^ phosphors.


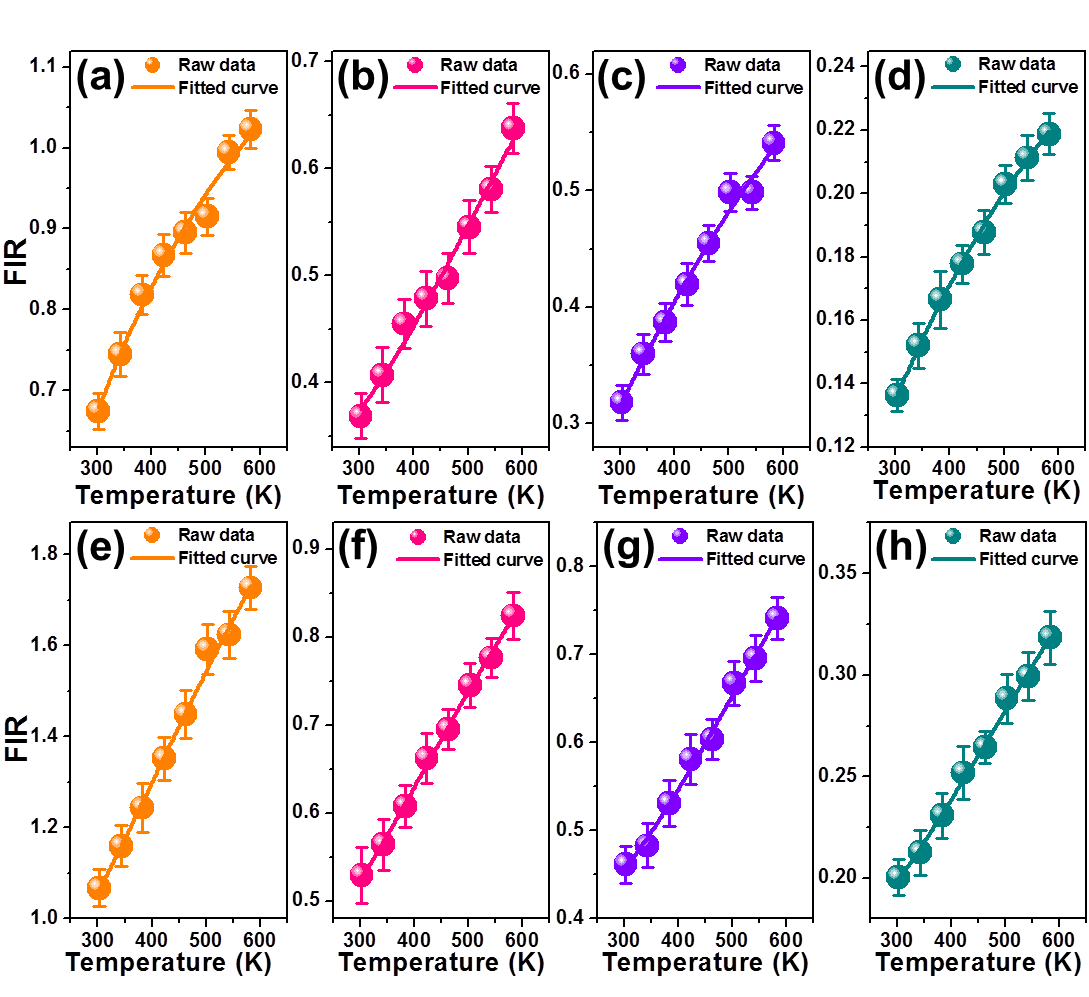


**Figure S10.** FIR values of the (a) Eu^2+^/^7^F_1_, (b) Eu^2+^/^7^F_2_, (c) Eu^2+^/^7^F_4_ and (d) total Eu^2+^/^7^F_J_ in the Li_2_CaSiO_4_:0.005Eu^2+^/Eu^3+^ phosphors as a function of temperature. FIR values of the (e) Eu^2+^/^7^F_1_, (f) Eu^2+^/^7^F_2_, (g) Eu^2+^/^7^F_4_ and (h) total Eu^2+^/^7^F_J_ in the Li_2_CaSiO_4_:0.04Eu^2+^/Eu^3+^ phosphors as a function of temperature.
